# History of Protein Data Bank Japan: standing at the beginning of the age of structural genomics

**DOI:** 10.1007/s12551-022-01021-w

**Published:** 2022-12-09

**Authors:** Genji Kurisu, Gert-Jan Bekker, Atsushi Nakagawa

**Affiliations:** Institute for Protein Research, Osaka University, 3-2 Yamadaoka, Suita, Osaka 565-0871 Japan

**Keywords:** Protein Data Bank, Protein Data Bank Japan, Computational biology, Bioinformatics

## Abstract

Prof. Haruki Nakamura, who is the former head of Protein Data Bank Japan (PDBj) and an expert in computational biology, retired from Osaka University at the end of March 2018. He founded PDBj at the Institute for Protein Research, together with other faculty members, researchers, engineers, and annotators in 2000, and subsequently established the worldwide Protein Data Bank (wwPDB) in 2003 to manage the core archive of the Protein Data Bank (PDB), collaborating with RCSB-PDB in the USA and PDBe in Europe. As the former head of PDBj and also an expert in structural bioinformatics, he has grown PDBj to become a well-known data center within the structural biology community and developed several related databases, tools and integrated with new technologies, such as the semantic web, as primary services offered by PDBj.

## Foundation of PDBj and the wwPDB

The Protein Data Bank (PDB) started with only seven entries in 1971 jointly by Brookhaven National Laboratory (BNL), USA and Cambridge Crystallographic Data Center, UK. At the time, all macromolecular structure data determined by structural biologists in the world were deposited to BNL, and uniformly curated and subsequently distributed as a single global archive, initially in analog form as punch cards, and later digitally on magnetic tapes, then CD-ROMs. However, in the late 1990s, the number of depositions increased dramatically mainly due to the development of new technologies; high-throughput beamlines at third generation synchrotron facilities, automated crystallization techniques, the spread of efficient techniques for production of recombinant proteins, improvements to high-field NMR machines, the emergence of ready-to-use software for structural analyses and the availability of high-speed, low-cost computers.

**Fig. 1:**
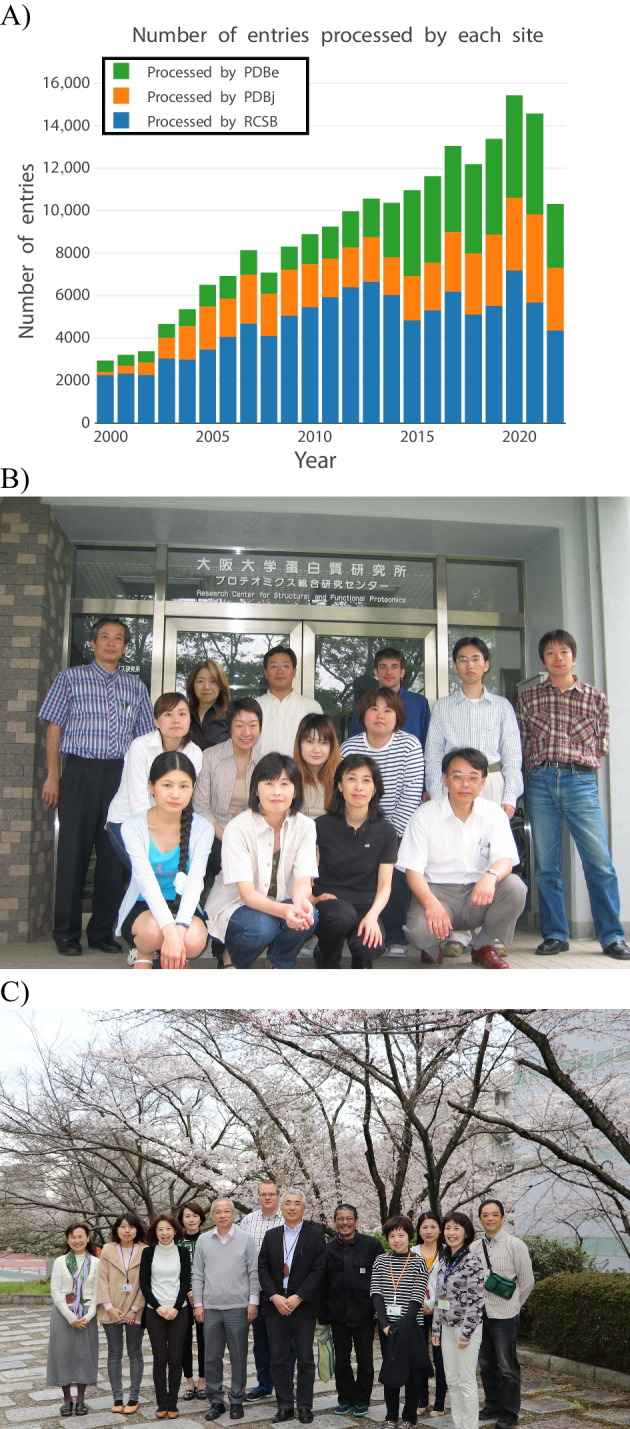
Changes to PDBj over the years. **a** Increase in depositions to PDBj. **b** First group photo of PDBj with Prof. Nakamura in 2003 and **c** the final one before he retired in 2017

Prof. Haruki Nakamura led his team of computational biologists at the Protein Engineering Research Institute, which was later renamed to the Biomolecular Engineering Research Institute, from 1987 and obtained original and influential results. In 1999, he was promoted to become a full Professor at the Institute for Protein Research, Osaka University, and started his database activities. Before he joined the IPR faculty, Dr. Masami Kusunoki and Dr. Genji Kurisu, one of this commentary’s authors, worked together with Prof. Joel L. Sussmann and his group from BNL-PDB and started a mirror service of the Brookhaven Protein Data Bank at Osaka University. Just after Prof. Nakamura’s move to IPR, the Protein Data Bank in the USA announced that they would move to the Research Collaboratory for Structural Bioinformatics (RCSB) and would be headed by Prof. Helen Berman of Rutgers University. As a new chair Professor, Prof. Nakamura invited Dr. Berman to IPR and had a constructive discussion with her on how to collaborate with RCSB-PDB. Together with Dr. Kusunoki, he started an official mirror service of RCSB-PDB and decided to fade-out the BNL mirror. However, with great foresight, Prof. Nakamura planned, budgeted, and implemented not just a mirror site, but also regional deposition (data-in) and unique data distribution (data-out) services in Asia. He and Dr. Kusunoki started a regional data-in service in 2000 with strong technical support from RCSB-PDB. He sent Mr. Takashi Kosada, the first lead annotator of PDBj, to RCSB-PDB to learn how to curate and annotate the various kinds of deposited macromolecular structural data. In 2000, PDBj processed 157 PDB entries, corresponding to about 5% of the yearly global deposited data. Now, 21 years later, this percentage has increased to 28% (Fig. 1a).

In 2001, Prof. Nakamura was awarded a big grant of 150,000,000 JPY per year to develop the Protein Data Bank activities in Japan from the BIRD (Institute for Bioinformatics Research and Development) project by the Japan Science and Technology agency over a period of 5 years. With this financial support, he managed the entire team (Fig. 1b and c) and named the organization Protein Data Bank Japan (PDBj). Even though PDBj is the smallest regional data center in both size and budget, it still has a local advisory board as part of the Joint-Usage/Research center activities of IPR, an activity that was authorized by the Ministry of Education, Culture, Sports, Science and Technology in Japan.

BMRB (Biological Magnetic Resonance Data Bank) is the experimental data archive of NMR founded in 1988 by Prof. John L. Markley, University of Wisconsin-Madison, USA. In 2002, Prof. Hideo Akutsu, an experimental NMR scientist at IPR, and Prof. Nakamura contacted Prof. Markley to discuss the establishment of a Japanese branch of the BMRB, originally called PDBj-BMRB, but recently rebranded to BMRBj, to cope with the rapid growth of NMR structural data in Japan at the time. Since then, BMRBj has processed about 10% of all BMRB entries, which were mainly deposited from Asian countries. Since Prof. Nakamura had been collaborating with NMR scientists for such a long time, he hosted the NMR validation taskforce and the NEF workshop meeting in Osaka in 2017, which established new descriptions of NMR experimental data along with stakeholders.

**Fig. 2:**
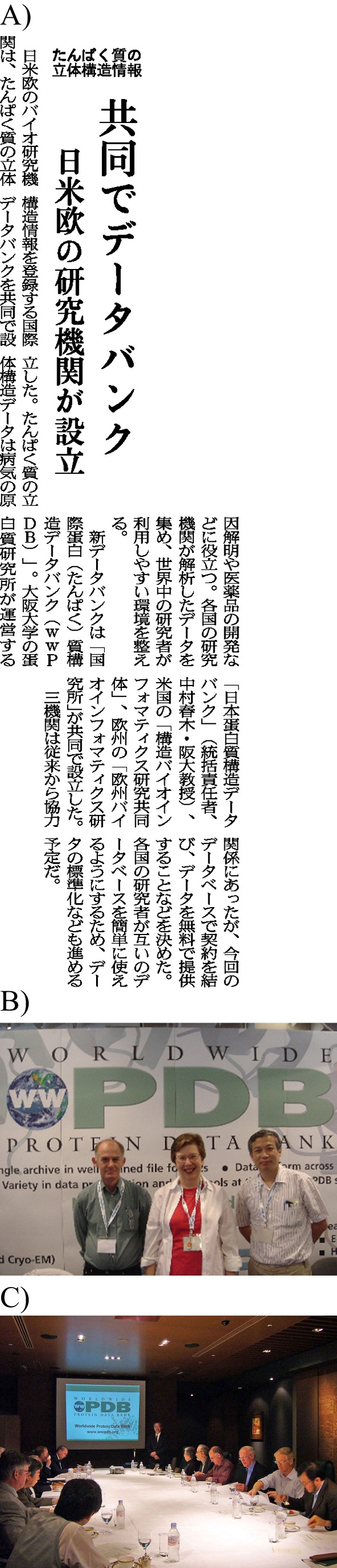
Snapshots of PDBj’s wwPDB-related activities. **a** Japanese newspaper article to announce the start of the wwPDB in the Nikkei Newspaper on December 2nd, 2003, shown with permission from the Nihon Keizai Shinbun Inc. The English translation of this article is as follows: “[Protein Data Bank, jointly managed by Japanese, European and American institutions, was established.] Research institutions in Japan, USA, and Europe have jointly established an international data bank for researchers to register protein structural data. The three-dimensional structural data of proteins are useful for elucidating the causes of diseases and developing new drugs. The data analyzed by the institutions in each country will be curated to create an environment that is easy for researchers all over the world. The new data bank is named the “worldwide Protein Data Bank.” It was jointly established by Protein Data Bank Japan, headed by Prof. Haruki Nakamura, and is co-hosted by the Institute for Protein Research, Osaka University, the Research Collaboratory for Structural Bioinformatics in the USA, and the European Bioinformatics Institute in Europe. The three organizations have been collaborating with each other for some time and have decided to conclude this into an agreement for the database and will continue to provide the data free of charge. They also have a plan to standardize the data in order to make it easier for researchers to use the database.” **b** Photo of the wwPDB PIs at IUCr-2005. **c** Photo of the third wwPDB-AC meeting (2006) held in Japan

Prof. Nakamura also played a crucial role in the foundation of the wwPDB. At the 19th congress of the International Union of Crystallography held in Geneva, Switzerland 2002, the three heads of the regional PDB data centers, Haruki Nakamura (PDBj), Janet Thornton (EBI), and Helen Berman (RCSB-PDB), were sitting together at the river side and had a discussion to form a unified, collaborative organization, which has now become known as the wwPDB. This meeting marked the beginning of the age of structural genomics, as more and more depositions were anticipated quite shortly, with the announcement of the formation of the wwPDB published in 2003 (Berman et al. 2003), which was also published in a Japanese newspaper (Fig. 2a). They agreed to keep a single global archive of the PDB and the archive were to be maintained jointly by the three regional data centers, who were later joined by the method-based data centers of BMRB and Electron Microscopy Data Bank (EMDB) (Burley et al. 2019). The wwPDB organization is based on the partnership between its members and the charter formalizing the agreement has continuously been amended and updated over the past two decades. The wwPDB also formed their own advisory committee nominated by its members, and wwPDB-AC meetings have been held annually in October or November since 2003, where Prof. Nakamura hosted the wwPDB-AC meetings of 2006, 2009, 2012, and 2015 in Japan (Fig. 2b and c). Thereby, the structural genomics community has been able to influence the policies of the wwPDB via the advisory committee. One of the changes was to increase the deposited experimental data, where in 2008, structure factor deposition became mandatory for crystallographic entries, while in 2010, NMR restraint file deposition related to the solution structure became mandatory. The largest change in the collaboration of the wwPDB partners came in 2015 with the introduction of a new unified worldwide deposition system called OneDep, simplifying deposition and annotation work, thereby paving the way for smooth operations with increasing number of depositions, especially from Asian countries.

## Unique services developed by Prof. Nakamura at PDBj

Over the years, Prof. Nakamura has been involved in the development of many tools and services at PDBj. During his time as the head of PDBj, most of the fundamental framework was developed. He started the eF-site and ProMode Elastic services as secondary databases derived from the PDB, which are still being maintained now. The eF-site service provides a database of electrostatic potentials’ surfaces pre-calculated, which is updated weekly together with the public release of new PDB entries (Kinoshita and Nakamura 2003). Alongside the initial release of eF-site was a new molecular viewer to visualize the structures and the surfaces, pdbjViewer or jV for short, which was a Java-based applet that could be embedded in web browsers of the time (Kinoshita and Nakamura 2004). The database and jV were developed by Dr. Kengo Kinoshita and the calculation of the electrostatic potential maps was based on Prof. Nakamura’s code that was first reported in 1987 (Nakamura and Nishida 1987). ProMode-Elastic is a database of normal mode analysis of PDB data (Wako et al. 2004), where the normal mode analysis is performed by a program called PDBETA, developed by Prof. Hiroshi Wako and Dr. Shigeru Endo (Wako and Endo 2013).

Another achievement led by Prof. Nakamura was the implementation and integration of the PDB with the semantic web together with Dr. Akira Kinjo, a project that has been funded by NBDC-JST since 2011. Currently, there are three official formats for PDB entries available: PDBx/mmCIF, PDBML (an XML based format), and RDF. The classical PDB format developed in the 1970s of the previous century for punch cards is no longer an official format, due to its limitations of a fixed number of columns (as it was designed for 80-column punch card readers) that have led to limitations in the of number of atoms, residues, and chains, in addition to severely limiting clear annotation of the structures. Here, PDBj was involved in the development of the PDBML format, where Dr. Nobutoshi Ito started working on an XML format as part of the BIRD project. At the same time, Dr. John Westbrook of RCSB-PDB independently developed his own XML format. These were finally integrated as the PDBML format (Westbrook et al. 2005). Later, PDBML was translated to the RDF format to link the PDB data to the semantic web and are published via our wwPDB/RDF service at https://rdf.wwpdb.org/ (Kinjo et al. 2012).

In addition, a relational database (RDB) called PDBj Mine was developed by Dr. Akira Kinjo, which was also based on the PDBML format and served as the main back-end database at PDBj (Kinjo et al. 2012), and was made accessible via REST and web interfaces to perform SQL searches. At that time, there was only limited annotation available within PDB entries, so PDBj annotators compiled additional annotation, which was made available via PDBj’s custom PDBMLplus format, mixing both PDB entry data and PDBj annotated data. Later, a revised version of the RDB, the Mine 2 RDB, was introduced, which was instead based on the PDBx/mmCIF data and segregated the PDBMLplus data into its own categories/tables to standardize the definitions used between the PDB data and the RDB, with the data also published in a JSON format called PDBx/mmJSON (Kinjo et al. 2017, 2018). Now, the RDB is also used directly to generate the content used on the individual entry pages on the PDBj website and we have also introduced a graphical helper service to help make it easier to perform some more advanced searches without requiring knowledge of SQL (Bekker et al. 2022). Finally, for users who would like to maintain their own copy of the RDB, we have made software available that updates their local copy of the RDB (https://gitlab.com/pdbjapan/mine2updater).

In 2007, PDBj developed the EM Navigator service (https://pdbj.org/emnavi/) (Kinjo et al. 2017), which is a website to explore 3DEM data in the EMDB, PDB, and later SASDB and was developed by Dr. Hirofumi Suzuki. At the time, only a small part of the entries in the PDB consisted of 3DEM structures, and only few users had the skills to view and manipulate 3DEM data, such as a 3D density map. Since typical 3DEM atomic models were quite difficult to view in web browsers at the time, the EM Navigator service used short movies to visualize the EMDB map data and/or PDB model data recorded from different orientations. More recently, PDBj developed additional services to enable structural-based searches of EM data. The first service is the Omokage search service, which is a shape similarity search service for 3D structures of macromolecules that compares the overall shape between registered structures or a user-submitted one (Suzuki et al. 2016). The second service is the gmfit service developed by Dr. Takeshi Kawabata, which also analyzes EM data and can be used to quickly fit 3D objects (either structures or density maps) onto each other using Gaussian mixture models (Kawabata 2008).

PDBj also developed several tools for the prediction of structural features of proteins. CRNPRED was developed by Dr. Akira Kinjo and can be used to predict characteristics of a protein such as secondary structure, contact numbers, and residue-wise contact orders from the amino acid sequence (Kinjo and Nishikawa 2006). A more recent tool is the HOMCOS service developed by Dr. Takeshi Kawabata, which can be used to model the quaternary structure of proteins based on homology modeling (Kawabata 2016). In addition, it can also be used to search for potential binding compounds given an amino acid sequence, or a set of binding proteins given a compound. The eF-seek service, which uses the surfaces and properties registered in PDBj’s eF-site secondary database, can be used to find proteins that have similar binding sites based on the shape and electrostatic properties of a query pocket (Kinoshita et al. 2007).

Molmil is a tool developed by PDBj (Bekker et al. 2016) that is not only used by structural biologists but also reaches towards the general population via PDBj’s outreach activities, where Molmil is used as a structural viewer to introduce the audience to protein structures. Initially, Prof. Nakamura wanted a molecular viewer to run on his tablet (an iPad), but Molmil has grown to be much more than just a molecular viewer for his tablet. Instead, Molmil was developed using the (at that time) latest web technology called WebGL, which is effectively an implementation of OpenGL for web browsers. Molmil launched in 2013 as a basic molecular viewer of PDB structures integrated in PDBj Mine’s entry pages, but in 2014 saw a major update, integrating it in many of PDBj’s web services, while also making the viewer available to load user-supplied structures in various formats. In subsequent years, additional functionality was added, including more advanced scripting and loading molecular dynamics trajectories. Although initially PDBj had used the Java-applet based jV viewer, technical changes in the operation of web browsers made it more difficult and eventually impossible to use Java-based applets like jV, thus making Molmil the molecular viewer of choice used by PDBj. It is available on every modern platform, from smartphones to headless servers, and it can also be used to visualize MD trajectories, create movies, and show protein structures in virtual reality environments (Bekker et al. 2022), a feature that has shown to also be useful for PDBj’s outreach activities.

**Fig. 3:**
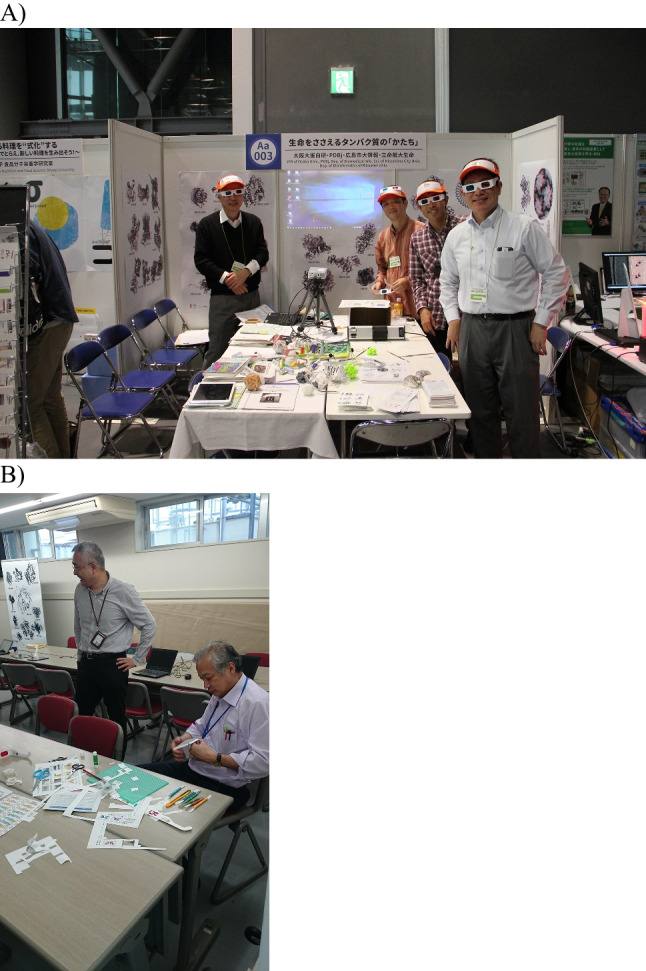
Prof. Haruki Nakamura at outreach activities organized by PDBj. **a** A group photo taken at “Science Agora 2016 in Tokyo.” **b** A snapshot photo taken at “Icho Sai, 2017 at IPR, Osaka University”

PDBj has had a wide range of outreach activities, both online, as well as offline. PDBj has long offered official Japanese translations of Dr. David Goodsell’s Molecule of the Month series on our website (https://numon.pdbj.org/mom/), whose originals are hosted in English at RCSB-PDB (https://pdb101.rcsb.org/motm/). Besides online resources, PDBj has also performed offline outreach activities. Although initially PDBj’s outreach activities focused on computational and experimental structural biologists by attending scientific conferences in Japan and educating the attendees via Luncheon seminars, in the past decade, PDBj has also expanded its outreach activities to the general public. At first, this was limited by attending Osaka University’s annual “Icho Sai” (Ginkgo Festival), where the University is opened to the general public who come to explore the university’s activities during Japan’s “Golden Week” in late April, early May (Fig. 3). Here, PDBj showcased its activities and introduced the world of structural biology to the general public, initially via printed images and physical models of proteins, while later, interactive interaction via laptops and tablets running Molmil was added. At this time, PDBj also started attending science fairs throughout Japan to bring our activities to the attention of an even wider general public and introduced visualization of protein structures via virtual reality. In recent years, this has grown even further and has now been consolidated into the PDBj Numon platform (https://numon.pdbj.org/) where, “numon” means beginner’s course in Japanese. The platform includes educational resources to help both students and non-specialists to learn about the 3D structures of biomolecules. Since the start of the pandemic, PDBj has also developed several feature pages that list all pertinent structural information related to COVID-19 to help both structural biologists and the general public get a better overview of the available entries related to COVID-19. These outreach pages have also been used in Japan by the press or broadcasting companies and have been cited in special documentaries. Thereby, PDBj has grown from a solely scientific organization, to an organization with roots within society, fostering the next generation of structural biologists.

## Closing words

When Prof. Nakamura started PDBj, he envisioned the emergence of a sprawling structural genomics community in Asia and viewed international collaboration to be crucial for the scientific community. In the past two decades, he has made significant contributions to the structural biology community, growing PDBj and the wwPDB into the organizations they are today. Since his retirement, we have done our best to honor his legacy and advance the structural genomics field. We are now collaborating with EMBL-EBI by mirroring the EMPIAR archive (Iudin et al. 2016) in Japan and also offer support to Asian depositors depositing to the EMPIAR archive. We have also developed two new novel archives at PDBj. The first ties in closely with Prof. Nakamura’s computational research with our new archive BSM-Arc (Bekker et al. 2020), which is an archive for computationally derived data. The second one ties in with data deposition of PDB data, where we have created a new archive called XRDa for depositing raw X-tal diffraction images for existing and new PDB entries. Then in 2020, we celebrated our 20th anniversary as the Asian hub of three-dimensional macromolecular structural data, a showcase to the efforts of Prof. Nakamura, whom without this would not have been possible. Thus, we greatly appreciate Prof. Nakamura’s enormous contribution to PDBj and the field of structural biology.
